# Supplementary material for: Coherent optical control of a superconducting microwave cavity via electro-optical dynamical back-action
Source: Nat Commun. 2023 Jun 24;14:3784. doi: 10.1038/s41467-023-39493-3 (PMC10290644; doi:10.1038/s41467-023-39493-3)
Supplement: Supplementary file 1 — Supplementary Information [file 41467_2023_39493_MOESM1_ESM.pdf]

Supplementary Information for
Coherent optical control of a superconducting microwave cavity via electro-optical dynamical back-action

Liu Qiu*,[†] Rishabh Sahu*, William Hease, Georg Arnold, and Johannes M. Fink[‡]

Institute of Science and Technology Austria, Am Campus 1, 3400 Klosterneuburg, Austria

(Dated: May 22, 2023)

CONTENTS

A. Theoretical model 2

1. Symmetric mode configuration 3
2. Stokes mode configuration 4
3. anti-Stokes mode configuration 4

B. Experimental Setup 5

C. Data Analysis 7

1. Susceptibility Reconstruction 7
2. Data Fitting of Stationary Dynamical Back-action 8
3. Data Fitting of Transient Dynamical Back-action 8

D. Instantaneous Excess Back-action 9

E. Excess Back-action 9

References 13

[†] liu.qiu@ist.ac.at

[‡] jfink@ist.ac.at

A. Theoretical model

The total interaction Hamiltonian of the multimode cavity electro-optical system is given by $\hat{H}_I = \hat{H}_{\rm eo} + \hat{H}_J$, where $\hat{H}_{\rm eo}/\hbar = g_0 \hat{a}_p^\dagger \hat{a}_s \hat{b} + g_0 \hat{a}_p^\dagger \hat{a}_{as} \hat{b}^\dagger + h.c.$, and $\hat{H}_J/\hbar = J_s \hat{a}_s^\dagger \hat{a}_{s,\rm tm} + J_{as} \hat{a}_{as}^\dagger \hat{a}_{as,\rm tm} + h.c.$. Here, $\hat{a}_s$, $\hat{a}_p$, $\hat{a}_{as}$ and $\hat{b}$ are the annihilation operators for the Stokes, pump and anti-Stokes optical modes and microwave mode, and g_0 is the vacuum electro-optical coupling rate. $\hat{a}_{s,\rm tm}$ and $\hat{a}_{as,\rm tm}$ are the annihilation operators for the corresponding TM mode of the Stokes and anti-Stokes mode.

We define the susceptibility of microwave or optical mode as,

$$\chi_j(\Omega) = \frac{1}{\kappa_j/2 - i\Omega}, \tag{S1}$$

where j is e or o for microwave or optical mode.

The strong optical *on-resonance* pump results in photon enhanced electro-optical coupling rate $g = \sqrt{\bar{n}_p} g_0$, with $\bar{n}_p$ the mean photon number of the pump mode. Scattered Stokes and anti-Stokes sidebands are located on the lower and upper side of the pump by Ω_e. In practice, the Stokes and anti-Stokes sidebands are detuned from the Stokes and anti-Stokes mode by $\delta_{\rm s}$ and $\delta_{\rm as}$, mostly due to FSR and Ω_e mismatch. In the case of on-resonance pumping and zero dispersion, we have $\delta_{\rm s} = -\delta_{\rm as}$. For simplicity, we assume the TM mode is of the same frequency of the corresponding Stokes or anti-Stokes mode.

We define the noise operators in the vector form, with the mode operator and input noise operator vectors,

$$\begin{aligned} D &= \left(\hat{a}_s, \hat{a}_s^\dagger, \hat{a}_{as}, \hat{a}_{as}^\dagger, \hat{a}_{s,\rm tm}, \hat{a}_{s,\rm tm}^\dagger, \hat{a}_{as,\rm tm}, \hat{a}_{as,\rm tm}^\dagger, \hat{b}, \hat{b}^\dagger\right)^T, \\ D_{\rm in} &= \left(\hat{a}_{s,\rm in}, \hat{a}_{s,\rm in}^\dagger, \hat{a}_{s,0}, \hat{a}_{s,0}^\dagger, \hat{a}_{as,\rm in}, \hat{a}_{as,\rm in}^\dagger, \hat{a}_{as,0}, \hat{a}_{as,0}^\dagger, \hat{a}_{s,\rm tm,vac}, \hat{a}_{s,\rm tm,vac}^\dagger, \hat{a}_{as,\rm tm,vac}, \hat{a}_{as,\rm tm,vac}^\dagger, \hat{b}_{\rm in}, \hat{b}_{\rm in}^\dagger, \hat{b}_0, \hat{b}_0^\dagger\right)^T, \end{aligned} \tag{S2}$$

with $\hat{a}_{j,\rm in}$ ($\hat{a}_{j,0}$) and $\hat{b}_{\rm in}$ ($\hat{b}_0$) the input (intrinsic) noise operator for optical and microwave modes, and $\hat{a}_{j,\rm tm,vac}$ the TM vacuum noise. In the rotating frame of the microwave resonance and Stokes and anti-Stokes sideband, we obtain the full dynamics of the intracavity mode field from quantum Langevin equations in the Fourier space,

$$\hat{D} = \mathbf{M} \cdot \mathbf{L} \cdot \hat{D}_{\rm in}, \tag{S3}$$

where

$$\mathbf{M} = \begin{pmatrix} \chi_o^{-1}(\delta_s+\Omega) & 0 & 0 & 0 & -iJ_s & 0 & 0 & 0 & 0 & ig \\ 0 & \chi_o^{-1}(\Omega-\delta_s) & 0 & 0 & 0 & iJ_s & 0 & 0 & -ig & 0 \\ 0 & 0 & \chi_o^{-1}(\delta_{\rm as}+\Omega) & 0 & 0 & 0 & -iJ_{\rm as} & 0 & ig & 0 \\ 0 & 0 & 0 & \chi_o^{-1}(\Omega-\delta_{\rm as}) & 0 & 0 & 0 & iJ_{\rm as} & 0 & -ig \\ -iJ_s & 0 & 0 & 0 & \chi_{o,\rm tm}^{-1}(\delta_s+\Omega) & 0 & 0 & 0 & 0 & 0 \\ 0 & iJ_s & 0 & 0 & 0 & \chi_{o,\rm tm}^{-1}(\Omega-\delta_s) & 0 & 0 & 0 & 0 \\ 0 & 0 & -iJ_{\rm as} & 0 & 0 & 0 & \chi_{o,\rm tm}^{-1}(\delta_{\rm as}+\Omega) & 0 & 0 & 0 \\ 0 & 0 & 0 & iJ_{\rm as} & 0 & 0 & 0 & \chi_{o,\rm tm}^{-1}(\Omega-\delta_{\rm as}) & 0 & 0 \\ 0 & ig & ig & 0 & 0 & 0 & 0 & 0 & \chi_e^{-1}(\Omega) & 0 \\ -ig & 0 & 0 & -ig & 0 & 0 & 0 & 0 & 0 & \chi_e^{-1}(\Omega) \end{pmatrix}^{-1} \tag{S4}$$

$$\mathbf{L} = \begin{pmatrix} \sqrt{\kappa_{o,\rm ex}} & 0 & \sqrt{\kappa_{o,0}} & 0 & 0 & 0 & 0 & 0 & 0 & 0 & 0 & 0 & 0 & 0 & 0 & 0 \\ 0 & \sqrt{\kappa_{o,\rm ex}} & 0 & \sqrt{\kappa_{o,0}} & 0 & 0 & 0 & 0 & 0 & 0 & 0 & 0 & 0 & 0 & 0 & 0 \\ 0 & 0 & 0 & 0 & \sqrt{\kappa_{o,\rm ex}} & 0 & \sqrt{\kappa_{o,0}} & 0 & 0 & 0 & 0 & 0 & 0 & 0 & 0 & 0 \\ 0 & 0 & 0 & 0 & 0 & \sqrt{\kappa_{o,\rm ex}} & 0 & \sqrt{\kappa_{o,0}} & 0 & 0 & 0 & 0 & 0 & 0 & 0 & 0 \\ 0 & 0 & 0 & 0 & 0 & 0 & 0 & 0 & \sqrt{\kappa_{o,\rm tm}} & 0 & 0 & 0 & 0 & 0 & 0 & 0 \\ 0 & 0 & 0 & 0 & 0 & 0 & 0 & 0 & 0 & \sqrt{\kappa_{o,\rm tm}} & 0 & 0 & 0 & 0 & 0 & 0 \\ 0 & 0 & 0 & 0 & 0 & 0 & 0 & 0 & 0 & 0 & \sqrt{\kappa_{o,\rm tm}} & 0 & 0 & 0 & 0 & 0 \\ 0 & 0 & 0 & 0 & 0 & 0 & 0 & 0 & 0 & 0 & 0 & \sqrt{\kappa_{o,\rm tm}} & 0 & 0 & 0 & 0 \\ 0 & 0 & 0 & 0 & 0 & 0 & 0 & 0 & 0 & 0 & 0 & 0 & \sqrt{\kappa_{e,\rm ex}} & 0 & \sqrt{\kappa_{e,0}} & 0 \\ 0 & 0 & 0 & 0 & 0 & 0 & 0 & 0 & 0 & 0 & 0 & 0 & 0 & \sqrt{\kappa_{e,\rm ex}} & 0 & \sqrt{\kappa_{e,0}} \end{pmatrix}. \tag{S5}$$

$\kappa_{j,0}$ and $\kappa_{j,\rm ex}$ correspond to the intrinsic loss and external coupling rate of mode j. We can obtain the effective susceptibility of microwave and optical mode $\chi_{j,\rm eff}(\Omega)$ from Eq. S3.

The output probing field can be obtained via input-output theorem, $\hat{a}_{j,\mathrm{out}} = \hat{a}_{j,\mathrm{in}} - \sqrt{\kappa_{j,\mathrm{ex}}}\hat{a}_j$. From the output field, we can obtain the incoherent output noise spectral density via Wiener–Khinchin theorem in different detection schemes. In this work, we focus on the coherent response of the multimode CEO system, where the amplitude reflection efficiency *in the lab frame* is given by,

$$S_{jj}(\Omega+\omega_j) = 1 - \eta_j \kappa_j \chi_{j,\mathrm{eff}}(\Omega), \tag{S6}$$

with $\eta_j = \kappa_{j,\mathrm{ex}}/\kappa_j$ the external coupling efficient for mode j. We define the spectral normalized reflection as the ratio of the reflection efficiency between pulse on and off,

$$R_j(\omega) = |S_{jj}(\omega)/S_{jj,\mathrm{off}}(\omega)|^2, \tag{S7}$$

with $S_{jj,\mathrm{off}}(\omega) = 1 - \kappa_{j,\mathrm{ex}}\chi_j(\Omega)$. Before the pulse is switched on, the absent back-action leads to $R_j(\omega) = 1$. During the pulse, the coherent and excess back-action leads to modification of $R_j(\omega)$. After the pulse, $R_j(\omega)$ restores to 1 if the repetition time is long enough. We note that, in case of *on-resonance microwave probing*, the normalized reflection

$$R_e(\Omega_e) = \left|\frac{1 - \kappa_{e,\mathrm{ex}}/((\kappa_e + \delta\kappa_e)/2 + i\delta\Omega_e)}{1 - 2\eta_e}\right|^2, \tag{S8}$$

is more susceptible to the microwave frequency shift $\delta\Omega_e$ compared to the linewidth change $\delta\kappa_e$.

1. *Symmetric mode configuration*

For ideal detunings ($\delta_s = \delta_{\mathrm{as}} = 0$), microwave effective susceptibility remains the same,

$$\chi_{e,\mathrm{eff}}(\Omega) = \chi_e(\Omega), \tag{S9}$$

due to evaded electro-optical dynamical back-action. In practice, excess back-action exists where $R_e(\Omega)$ slightly deviates from 1, as shown in the main text. Despite the absent dynamical back-action to the microwave mode, the optical susceptibility around the Stokes and anti-Stokes modes are changed, which takes the form,

$$\chi_{o,\mathrm{s/as}}(\Omega) = \frac{1}{\chi_o(\Omega)^{-1} \mp g^2/(\chi_e(\Omega)^{-1} \pm g^2\chi_o(\Omega))}. \tag{S10}$$

In Fig. S1 , we show the theoretical curves of the optical coherent response in the symmetric case ($J_{\mathrm{s/as}} = 0$) at different C. For low C, $\chi_{o,\mathrm{s}}(\Omega)$ and $\chi_{o,\mathrm{as}}(\Omega)$ show similar behavior to electro-optically induced absorption (EOIA) and transparency (EOIT), due to the constructive and destructive interference between the probe field and the electro-optical interaction induced field. As C increases, both the Stokes and anti-Stokes mode probing response

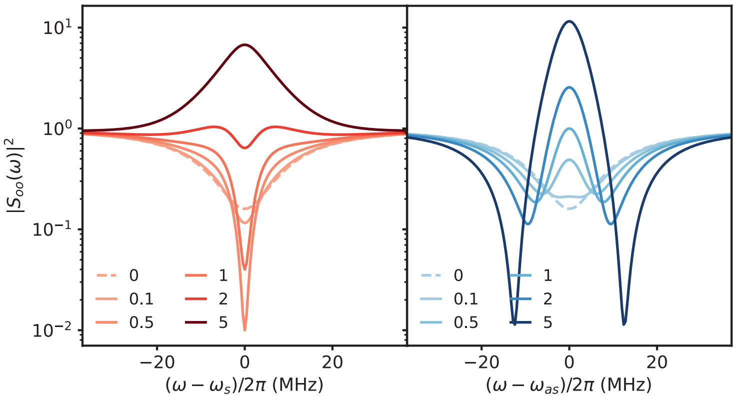

FIG. S1. **Theoretical coherent optical response in the symmetric case at different C.** The left panel corresponds to the optical probing around the Stokes mode, while the right panel corresponds to optical probing around the anti-Stokes mode. (Parameters: $\kappa_o/2\pi = 30$MHz, $\eta_o = 0.3$, $\kappa_e/2\pi = 10$MHz)

deviate from typical EOIA and EOIT behavior. For example, the optical reflection coefficient can even exceed unitary around resonance for anti-Stokes mode probing. Even in the symmetric case, the complex optical response of the multimode CEO system can be utilized for dispersion engineering of the probing field. At large C (e.g. $C \gg 2$), the symmetric multimode CEO system can function as a broadband electro-optical parametric amplifier for both Stokes and anti-Stokes signals.

2. *Stokes mode configuration*

In the Stokes case, i.e. $J_s = 0$, effective microwave susceptibility is given by,

$$\chi_{e,s}(\Omega) = \frac{1}{\chi_e^{-1}(\Omega) + g^2\left(\frac{\chi_o(\Omega+\delta_{\rm as})}{1+J_{\rm as}^2\chi_o(\Omega+\delta_{\rm as})\chi_{o,\rm tm}(\Omega+\delta_{\rm as})} - \chi_o(\Omega+\delta_s)\right)}. \tag{S11}$$

As shown in the theoretical curves in Fig. 2 and 3, dynamical back-action results in microwave frequency shift (optical-spring effect) and linewidth decrease,

$$\begin{aligned}
\delta\Omega_e &= -\frac{4g^2\delta_s}{\kappa_o^2+4\delta_s^2} + \frac{4\delta_{as}g^2\left(\kappa_{o,\rm tm}^2 - 4J_{\rm as}^2 + 4\delta_{as}^2\right)}{8J_{\rm as}^2\left(\kappa_o\kappa_{o,\rm tm} - 4\delta_{as}^2\right) + \left(4\delta_{as}^2+\kappa_o^2\right)\left(\kappa_{o,\rm tm}^2 + 4\delta_{as}^2\right) + 16J_{\rm as}^4}\\
\delta\kappa_e &= -\frac{4g^2\kappa_o}{\kappa_o^2+4\delta_s^2} + \frac{4g^2\left(\kappa_{o,\rm tm}\left(\kappa_o\kappa_{o,\rm tm}+4J_{\rm as}^2\right) + 4\delta_{as}^2\kappa_o\right)}{8J_{\rm as}^2\left(\kappa_o\kappa_{o,\rm tm} - 4\delta_{as}^2\right) + \left(4\delta_{as}^2+\kappa_o^2\right)\left(\kappa_{o,\rm tm}^2 + 4\delta_{as}^2\right) + 16J_{\rm as}^4},
\end{aligned} \tag{S12}$$

where our experiment are in the normal dissipation regime, i.e. $\kappa_o \gg \kappa_e$.

In the case of ideal detuning, i.e. $\delta_s = \delta_{as} = 0$,

$$\begin{aligned}
\chi_{e,s}(\Omega) &= \frac{1}{\chi_e^{-1}(\Omega) - g^2\chi_o(\Omega)\left(1 - r_{\rm as}(\Omega)\right)}\\
\chi_{o,\rm s}(\Omega) &= \frac{1}{\chi_o^{-1}(\Omega) - g^2\chi_e(\Omega)/(1+g^2\chi_e(\Omega)\chi_o(\Omega)r_{\rm as}(\Omega))},
\end{aligned} \tag{S13}$$

where $r_{\rm as}(\Omega) = [1 + J_{as}^2\chi_o(\Omega)\chi_{o,\rm tm}(\Omega)]^{-1} < 1$ is the anti-Stokes and Stokes scattering rate ratio.

In the case of $4J_{\rm as}^2 \gg \kappa_o\kappa_{o,\rm tm}$, where the anti-Stokes scattering is completely suppressed, we obtain,

$$\begin{aligned}
\chi_{e,s}(\Omega) &= \frac{1}{\chi_e(\Omega)^{-1} - g^2\chi_o(\Omega)}\\
\chi_{o,\rm s}(\Omega) &= \frac{1}{\chi_o(\Omega)^{-1} - g^2\chi_e(\Omega)},
\end{aligned} \tag{S14}$$

which is *symmetric under interchange of microwave and the Stokes mode.*

As seen from the red curves in Fig. S2, the microwave response shows *effective narrowing in the normal dissipation regime (upper left), while EOIA in the reversed dissipation regime (upper right).* The optical response around the Stokes mode shows *EOIA in the normal dissipation regime (lower left), while effective narrowing in the reversed dissipation regime (lower right).* The asymmetric multimode CEO system can be adopted for "fast light" of optical (microwave) probing field in the normal (reversed) dissipation regime, with reduced group delay.

3. *anti-Stokes mode configuration*

In the anti-Stokes case, i.e. $J_{\rm as} = 0$, effective microwave susceptibility is given by,

$$\chi_{e,\rm as}(\Omega) = \frac{1}{\chi_e(\Omega)^{-1} + g^2\left(\chi_o(\Omega+\delta_{\rm as}) - \frac{\chi_o(\Omega+\delta_s)}{1+J_s^2\chi_o(\Omega+\delta_s)\chi_{o,\rm tm}(\Omega+\delta_s)}\right)} \tag{S15}$$

As shown in the theoretical curves in Fig. 2 and 3, the dynamical back-action results in optical-spring effect and effective microwave linewidth increase,

$$\begin{aligned}
\delta\Omega_e &= \frac{4g^2\delta_{\rm as}}{4\delta_{\rm as}^2+\kappa_o^2} - \frac{4g^2\delta_s\left(\kappa_{o,\rm tm}^2 - 4J_s^2 + 4\delta_s^2\right)}{8J_s^2\left(\kappa_o\kappa_{o,\rm tm} - 4\delta_s^2\right) + \left(\kappa_o^2+4\delta_s^2\right)\left(\kappa_{o,\rm tm}^2+4\delta_s^2\right) + 16J_s^4}\\
\delta\kappa_e &= \frac{4g^2\kappa_o}{4\delta_{\rm as}^2+\kappa_o^2} - \frac{4g^2\left(\kappa_{o,\rm tm}\left(\kappa_o\kappa_{o,\rm tm}+4J_s^2\right) + 4\kappa_o\delta_s^2\right)}{8J_s^2\left(\kappa_o\kappa_{o,\rm tm} - 4\delta_s^2\right) + \left(\kappa_o^2+4\delta_s^2\right)\left(\kappa_{o,\rm tm}^2+4\delta_s^2\right) + 16J_s^4},
\end{aligned} \tag{S16}$$

as our experiment is in the normal dissipation regime, i.e. $\kappa_o \gg \kappa_e$.

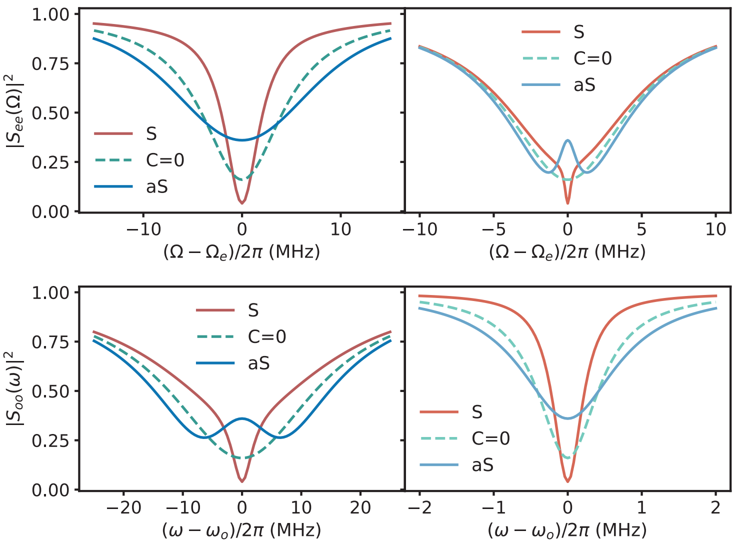

FIG. S2. **Theoretical coherent multimode electro-optical dynamical back-action in normal and reversed dissipation regime.** The left two panels correspond to the normal dissipation regime with $\kappa_o \gg \kappa_e$. (Parameters: $\kappa_o/2\pi = 30$MHz, $\eta_o = 0.3$, $\kappa_e/2\pi = 10$MHz, $\eta_e = 0.3$, and $C = 0.5$) The red and blue solid curves correspond to the Stokes and anti-Stokes case with $C = 0.5$, while the dashed curves correspond to the original reflection coefficient with $C = 0$. The right two panels correspond to the reversed dissipation regime with $\kappa_o \ll \kappa_e$. Different curves are similar to those in the left panels. (Parameters: $\kappa_o/2\pi = 1$MHz, $\eta_o = 0.3$, $\kappa_e/2\pi = 10$MHz, $\eta_e = 0.3$, and $C = 0.5$)

In the case of ideal detuning, i.e. $\delta_s = \delta_{\rm as} = 0$,

$$\begin{aligned} \chi_{e,\rm eff}(\Omega) &= \frac{1}{\chi_e^{-1}(\Omega) + g^2\chi_o(\Omega)\left(1 - r_{\rm s}(\Omega)\right)} \\ \chi_{o,\rm as}(\Omega) &= \frac{1}{\chi_o^{-1}(\Omega) + g^2\chi_e(\Omega)/(1 - g^2\chi_e(\Omega)\chi_o(\Omega)r_{\rm s}(\Omega))}, \end{aligned} \tag{S17}$$

where $r_s(\Omega) = [1 + J_s^2\chi_o(\Omega)\chi_{o,\rm tm}(\Omega)]^{-1} < 1$ is the Stokes and anti-Stokes scattering rate ratio. When the anti-Stokes scattering is fully suppressed ($4J_s^2 \gg \kappa_o\kappa_{o,\rm tm}$), we obtain,

$$\begin{aligned} \chi_{e,\rm as}(\Omega) &= \frac{1}{\chi_e(\Omega)^{-1} + g^2\chi_o(\Omega)} \\ \chi_{o,\rm as}(\Omega) &= \frac{1}{\chi_o(\Omega)^{-1} + g^2\chi_e(\Omega)}, \end{aligned} \tag{S18}$$

which is *symmetric under interchange of microwave and the anti-Stokes mode.*

As seen from the blue curves in Fig. S2, the microwave response shows *effective broadening in the normal dissipation regime (upper left), while EOIT in the reversed dissipation regime (upper right).* The optical response around the anti-Stokes mode shows *EOIT in the normal dissipation regime (lower left), while effective broadening in the reversed dissipation regime (lower right).* The asymmetric multimode CEO system can be adopted for "slow light" of optical (microwave) probing field in the normal (reversed) dissipation regime, with increased group delay. For simplicity, we assume the same cavity coupling coefficient 0.3 for both microwave and optical modes in the theoretical calculations in Fig. S2.

B. Experimental Setup

The experimental setup consists of optics preparation, microwave preparation, CEO device in the dilution fridge, optics and microwave detection, and data acquisition. More details of the experimental setup are shown in Fig. S3.

The normalized reflections of the optical modes as shown in the main text are fitted using coupled mode theory, with results shown in Table S1. The mode with largest splitting, i.e. mode 4, is adopted as the split mode in the asymmetric case. We note that, the obtained detunings of TE and TM mode in mode 4 are quite similar. For this reason, we

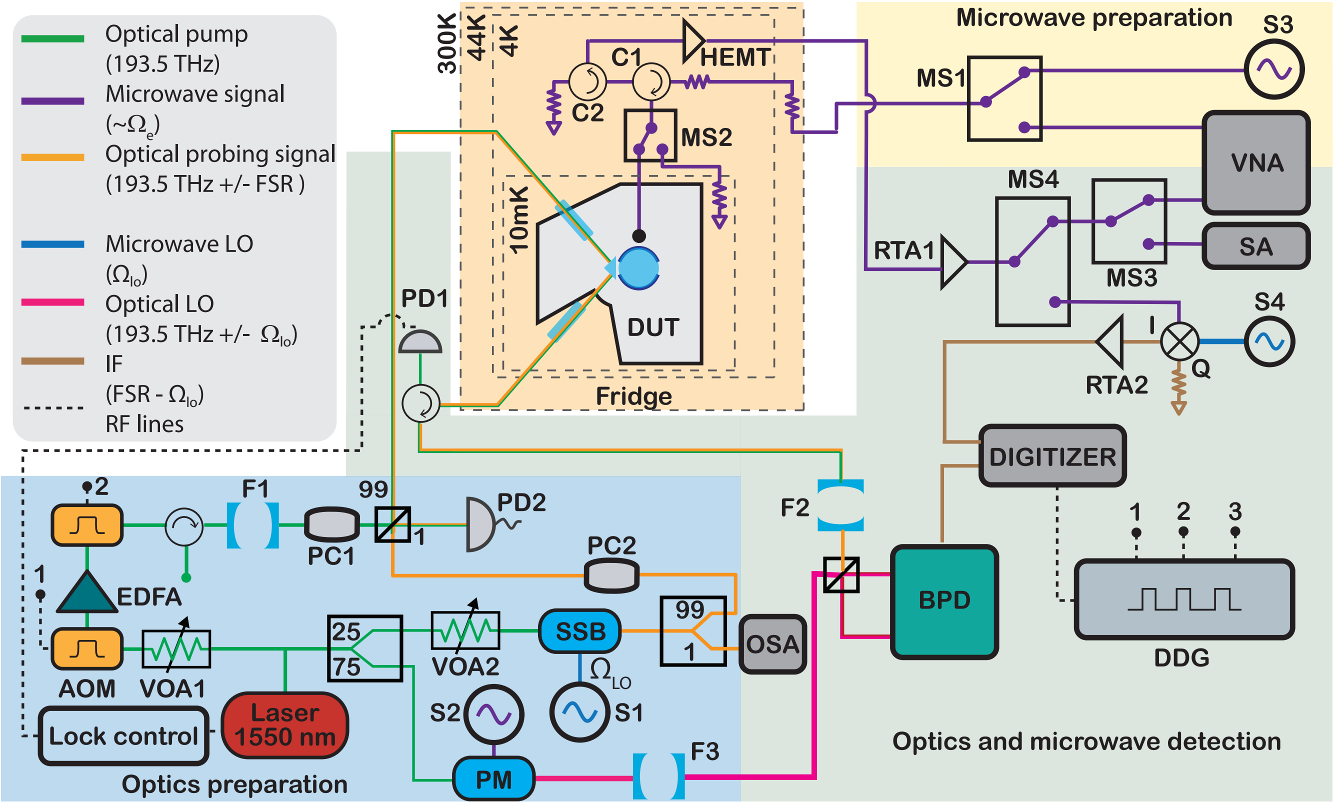

FIG. S3. **Experimental Setup**. On the optical side, laser light from Toptica CTL 1550 is divided into two equal parts - one for the optical pump, and the other for the optical signal and local oscillator (LO). The optical pump (left) passes through a variable optical attenuator (VOA1), and is then sent to an acousto-optic modulator (AOM1). The AOM is pulsed using a digital delay generator (DDG). The generated optical pulses are amplified by an Erbium-doped fiber amplifier (EDFA). Output of the EDFA is sent to another AOM2, which is connected to DDG and is used to suppress the broad-band amplified spontaneous emission (ASE) noise from the EDFA. The optical pump pulse is further filtered with an on-resonance filter cavity F1 (50 MHz linewidth with $\sim$15 GHz FSR), which is frequency locked to the Toptica laser. The signal arm is first divided in two parts, i.e. the optical signal and LO. 25% of the light in signal arm is used to produce the optical probing signal using a single sideband modulator (SSB), continuously monitored by an optical spectrum analyzer (OSA). The 75% light on the right side of the laser is used to produce the optical LO via a phase modulator (PM). The PM is operated via a microwave source S2 for efficient sideband generation and carrier suppression. Optical LO is sent to a filter cavity F3 to suppress the spurious optical tones from the PM. The pump pulse together with the optical probing signal are sent to the dilution refrigerator (DR). In the DR, light is focused via a gradient-index (GRIN) lens on the surface of a prism and coupled to the optical whispering gallery mode resonator (WGMR) via evanescent coupling. Fiber polarization controllers PC1 and PC2 are adjusted for efficient TE mode coupling of the optical WGMR. The output light from the optical WGMR is collected by the grin lens and is sent to filter F2 (50 MHz linewidth with $\sim$40 dB suppression) to reject the strong optical pump. The reflected optical pump from cavity F2 is used to lock the laser to the optical pump mode and is partially captured by photo-diode PD1 via the circulator C4. The transmitted optical probing signal and optical LO are sent directly to the optical balanced heterodyne setup with a balanced photo-detector (BPD). The output signal is amplified via a room temperature amplifier RTA1 before being sent to a digitizer. On the microwave side, the probing microwave signal is sent from microwave source S3 (or from the VNA for microwave mode spectroscopy) to the fridge input line via the microwave switch (MS1). The input line is attenuated by 60dB with cryogenic attenuators distributed between 3 K and 10 mK to suppress room temperature microwave noise. Circulator C1 redirects the reflected tone from the cavity to amplified output line, while C2 redirects noise coming in from the output line to a matched 50 Ω termination. Microwave switch MS2 allows to swap the device under test (DUT) for a temperature $T_{50\Omega}$ controllable load. The output line is amplified by a HEMT-amplifier at 3 K and a low noise amplifier (LNA) at room temperature. The output line is connected to switch MS4 and MS3, to select between SA, VNA or down-conversion using MW LO S4 for digitizer measurement.

assume the same frequency for the TM mode to its corresponding TE mode in the main text. Depending on the specific pump configuration, the microwave cavity frequency is adjusted to match the optical pump and probe mode separation, as shown in Table S3. The complete information regarding the frequency separation between the optical modes are shown in Table S2. The imperfect detunings between the Stokes and anti-Stokes modes are considered

Modes	$\kappa_o/2\pi$ (MHz)	$\kappa_{o,\mathrm{ex}}/2\pi$ (MHz)	$\delta_o/2\pi$ (MHz)	$\kappa_{o,\mathrm{tm}}/2\pi$ (MHz)	$\delta_{o,\mathrm{tm}}/2\pi$ (MHz)	$J/2\pi$ (MHz)
4	34.6	8.9	-17.8	7.6	-18.5	26
5	24.7	9.8	5	17.4	28.3	13
6	24.3	9.2	-3.9	30	-18.7	10

TABLE S1. **Fitted parameters for the split modes.** κ_o and $\kappa_{o,\mathrm{tm}}$ correspond to the total loss rate of the TE and corresponding TM mode. δ_o and $\delta_{o,\mathrm{tm}}$ correspond to the cavity detuning of TE and corresponding TM mode to the main dip of the splitted mode, which is centered to zero in Fig. 1(c).

Modes	1 and 2	2 and 3	3 and 4	4 and 5	5 and 6
Separation	8.799GHz	8.799GHz	8.791GHz	8.817GHz	8.795GHz

TABLE S2. **Calibrated frequency separation of the adjacent optical modes, shown as the distance between the main dip of each optical modes.**

in the calculation of dynamical back-action using full theoretical model in the main text, especially regarding the optical-spring effect since it is sensitive to detunings [cf. Eq. S12 and Eq. S16].

For the coherent response experiments in the pulsed regime, we send short optical pump pulse ($\tau \sim 50\mathrm{ns} - 2\mu s$) to the CEO device, while keeping the weak microwave or optical probing field on. The optical pump pulses are triggered at rate of 100 Hz for all the experiments, except for the Stokes case (2Hz). We sweep the frequency around the probing mode to reconstruct the full microwave or optical response. For each frequency, the pulses are repeated 2500 times. In addition, we sweep the pump pulse power to investigate the power dependence of the dynamical back-action with peak power $\sim$ 500mW. The RF signal from the balanced heterodyne detection of the optical probing field and the frequency down-converted microwave signal are recorded by a digitizer. In our experiments, both optical and microwave LO are detuned by 40MHz from the probing signal frequency. All the dynamical back-action data are taken from the time domain traces at 1GS/s sampling rate for different mode and probing configurations, except for the delayed excess back-action data shown in Fig. 4(b) and (c) of the main text, which is taken by the SA in the zero-span mode.

C. Data Analysis

In this work, we focus on the coherent response of the multimode CEO device. Here we show the detailed procedure for the data analysis.

1. *Susceptibility Reconstruction*

The basic principle for susceptibility reconstruction of the CEO device is shown in the main text. The spectral normalized reflection for the probing field is defined as,

$$R_j(\omega) = |S_{jj}(\omega)/S_{jj,\mathrm{off}}(\omega)|^2, \tag{S19}$$

where $S_{jj}(\omega)$ and $S_{jj,\mathrm{off}}(\omega)$ are the reflection coefficient of the probing field j with pulse on and off in the lab frame. For simplicity, we approximate the reflection with pulse off $S_{jj,\mathrm{off}}(\omega)$ to $S_{jj}(\omega)|_{t=0}$, i.e. the normalized reflection before the pulse arrives.

In the experiments, the weak coherent RF signal from the down-converted microwave and optical field $I_j(t)$ is fixed at 40MHz, more than 10 dB above the noise floor, due to the low noise amplification using HEMT amplifier or optical

Pump Config.	Probing Mode	Pump Mode	Probe Mode	MW Frequency
Sym.	Stokes	2	1	8.799 GHz
Sym.	anti-Stokes	2	3	8.799 GHz
Stokes	Stokes	3	2	8.799 GHz
anti-Stokes	anti-Stokes	5	6	8.795 GHz

TABLE S3. **Measurement details for different mode and probing configurations.** The pump mode and probe mode indexes are given for each probing configurations. Microwave frequency is adjusted accordingly to match the pump and probing mode separation.

balanced heterodyne detection. Here we only focus on the output power in the detection, as the phase information are washed out due to the drift between pulses. For a phase sensitive coherent response measurement, e.g. VNA, additional weak optical pulses can be applied to obtain an insitu phase correction in each trigger.

We perform digital down-conversion (DDC) of the time-domain data at 40MHz and reconstruct the normalized reflection coefficient over time for different probe field frequencies,

$$R_j(\Omega + \Omega_{\mathrm{LO},j}) = \frac{\bar{P}_{\mathrm{out},j}(\Omega)}{\bar{P}_{\mathrm{out},j}(\Omega)|_{t=0}}, \tag{S20}$$

with $\Omega_{\mathrm{LO},j}$ the LO frequency and $\bar{P}_{\mathrm{out},j}$ the averaged power of the RF field from DDC. This avoids the complicated system calibration due to the frequency dependence on the input and detection sides, especially on the optical side due to the pump filter (F2).

2. Data Fitting of Stationary Dynamical Back-action

As our multimode CEO device is in the normal dissipation regime, i.e. $\kappa_o \gg \kappa_e$, microwave frequency shift and linewidth change result in an effective susceptibility,

$$\chi_{e,\mathrm{eff}}(\Omega) = \frac{1}{(\kappa_e + \delta\kappa_e)/2 - i(\Omega - \delta\Omega_e)}, \tag{S21}$$

where $\delta\kappa_e$ and $\delta\Omega_e$ are the linewidth and frequency change of microwave mode. As mentioned in SI A, the on-resonance microwave probing is more susceptible to microwave frequency shift. Considering the complex back-action dynamics, we don't adopt full model of coherent electro-optical dynamical back-action for the microwave response fitting.

On the optical side, we adopt full DBA model for the coherent response fitting, including imperfect detunings. For the stationary dynamical back-action, we perform a joint fit of the coherent microwave and optical response at the steady regime of the pulse for all the powers, with microwave linewidth, microwave external coupling rate, optical linewidth, optical external coupling rate as shared parameters as shown in Fig. S7 to S10. The resulted fitting parameters, including the imperfect detunings are adopted to give the theoretical curves in Fig. 2 and 3 in the main text.

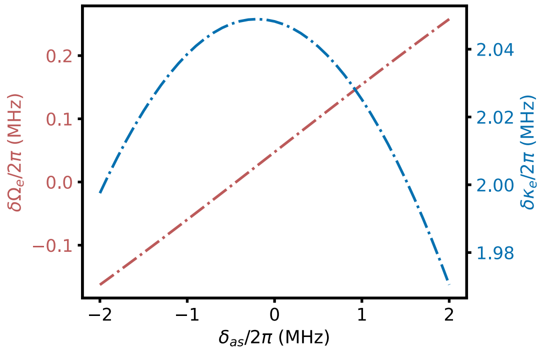

FIG. S4. **Electro-optical back-action to the microwave mode for the anti-Stokes case.** Estimated optical spring effect ($\delta\Omega_e$) and microwave linewidth change ($\delta\kappa_e$) versus the detuning uncertainty ($\delta_{\mathrm{as}} = \omega_{\mathrm{as}} - \omega_p - \Omega_e$) at $C = 0.2$.

In Fig. 2(b), we observe a discontinuous optical spring effect versus C in the anti-Stokes case ($\omega_p = \omega_5$), especially around $C \sim 0.2$. This might be due to the detuning uncertainties in the experiments, where we show an estimated microwave frequency and linewidth change due to imperfect detuning δ_{as} at $C = 0.2$ in Fig. S4. Minuscule optical spring effect exists for $\delta_{as} = 0$, which is due to the asymmetric Stokes modes (ω_4) [cf. Tab. S2 and S1]. We note that, such detuning uncertainty is also observed in the transient dynamical back-action [cf. Fig. S5].

3. Data Fitting of Transient Dynamical Back-action

For the instantaneous dynamical back-action, the fitting of microwave and optical response are performed separately. On the microwave side, for measurements with given pulse power we perform a joint fit of the coherent response over

the pump pulse (from the beginning of the pulse till $\sim 3\mu s$ after the pulse), to capture the delayed excess back-action due to the pump pulse. On the optical side, we only focus on the pulsed regime, where C over time is obtained as shown in Fig. S5(a), since the optical coherent response restores instantaneously after the pulse off. We note that, to capture perfectly the temporal dynamics on the optical side, *a free parameter* $\delta = \Omega_e - \mathrm{FSR}$ *needs to be introduced in the fitting*, which indicates pump pulse induced FSR or microwave frequency change during the pulse, as explained in the next section (SI D). The resulted fitting parameters are adopted to give the theoretical curves during the pulse in Fig. 4(a). The obtained microwave frequency and linewidth change after the pulse are shown in Fig. S6.

D. Instantaneous Excess Back-action

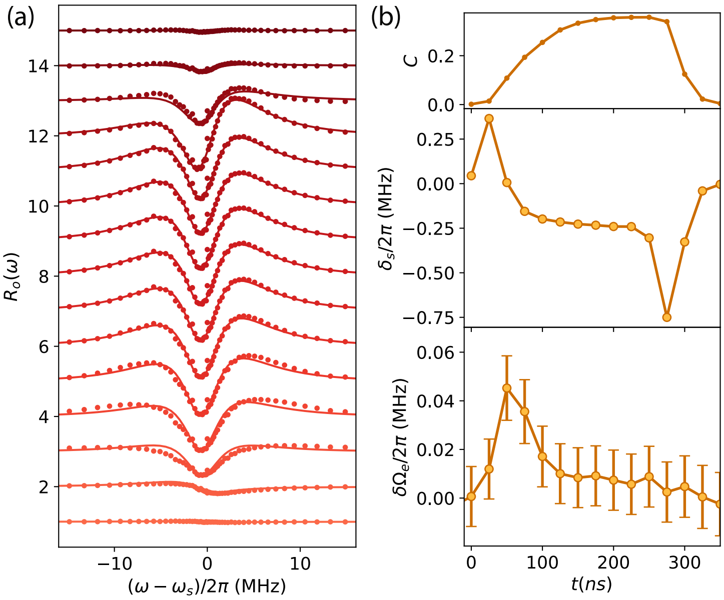

FIG. S5. **Instantaneous excess back-action over the pulse for the symmetric case. a,** Displaced instantaneous optical normalized reflection $R_o(\omega)$ around the Stokes mode during the pulse, with corresponding fitting curves. **b,** Upper panel, fitted C from optical response over the pulse. Middle panel, fitted δ_s, i.e. $\Omega_e - \mathrm{FSR}$ from optical response over the pulse. Lower Panel, fitted $\delta\Omega_e$ from microwave response over the pulse. Error bars indicate two standard deviations.

In Fig. S5(a), we show the displaced coherent optical response $R_o(\omega)$ over the pulse with corresponding fitting curves. During the loading and unloading of the optical pump, $R_o(\omega)$ is not symmetric around the Stokes mode resonance, which indicates frequency mismatch between Ω_e and the optical mode separation, i.e. $\delta_s = \Omega_e - \mathrm{FSR} \neq 0$. In Fig. S5(b), we show the fitted C and δ_s in the upper and middle panel, while $\delta\Omega_e$ in the lower panel. We note that, a detuning change of $\sim$ 1MHz arises during the loading and unloading of the pulse. The exact reason for the detuning change requires further exploration. It can be attributed to either optical FSR change, e.g. due to photo-refractive effect [S1] or dissipative feedback [S2], or intrinsic microwave frequency change, e.g. due to quasi-particles [S3, S4].

E. Excess Back-action

In Fig. 4(b) of the main text, we show the excess delayed back-action after the pulse with resonant ($\Omega_e = \mathrm{FSR}$) and off-resonant ($\Omega_e \neq \mathrm{FSR}$) comparison, for the symmetric mode configuration ($J_{\mathrm{s/as}} = 0$). In Fig. S6, we show the excess back-action to the microwave mode of the symmetric case in Fig. 4(a), with extracted microwave frequency and linewidth change. Figure S6(a) shows the 2D plot of $R_e(\Omega)$ during the pulse, where the large color contrast around resonance indicates microwave frequency shift [cf. Eq. S8]. Even for low C, the excess delayed back-action is rather evident after the pulse is off, where we observe a dramatic microwave frequency shift at $t_{\mathrm{ex}} \sim 1300ns$, despite of the absent optical pump pulse. Figure S6(b) shows the fitted frequency and linewidth change during the pulse. We note that, for high C, the microwave linewidth decreases when the pulse arrives, which is consistent with Fig. 4(a) (middle panel) and the microwave reflection decrease in Fig. 1(e) (upper left). As C increases, the color contrast decreases,

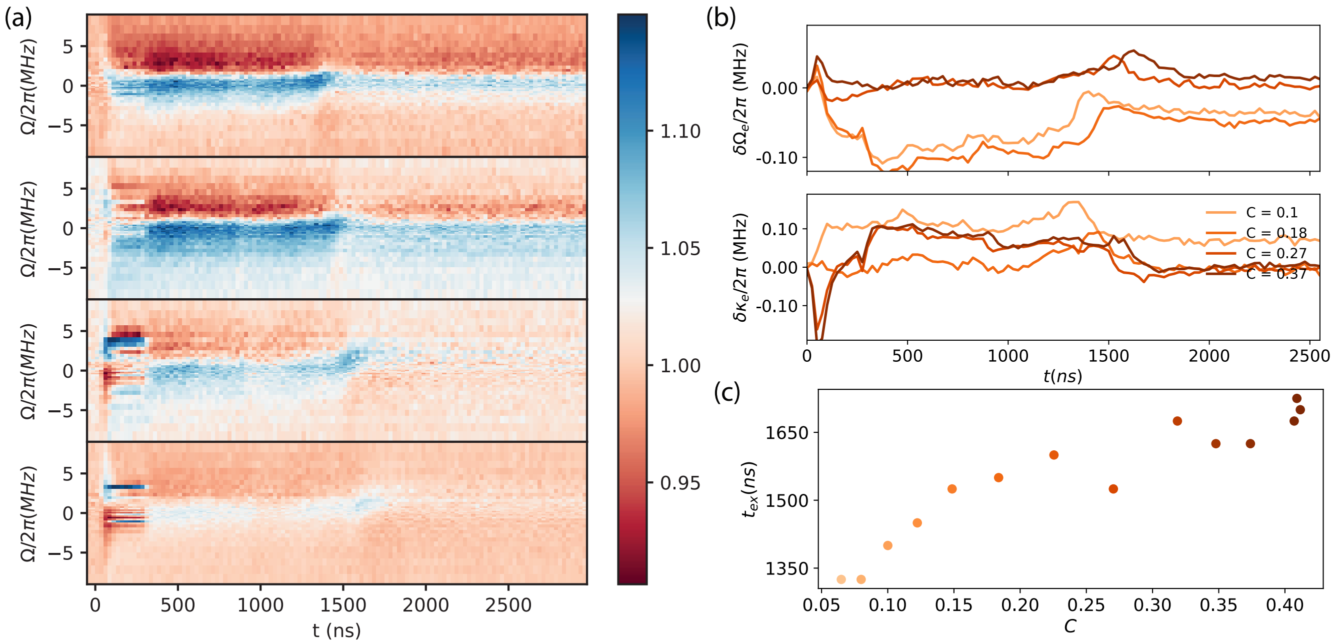

FIG. S6. **Excess back-action to the microwave mode for the symmetric case.** **a**, Normalized reflection coefficients $R_e(\Omega)$ over the pump pulse (from the top to bottom) for C of 0.1, 0.18, 0.27, and 0.37. **b**, Fitted microwave frequency and linewidth change after the pulse from (a). Different color corresponds to different C. **c**, Extracted C dependent $t_{\rm ex}$, i.e. the transition time for the excess back-action after the pulse is off.

while $t_{\rm ex}$ slowly increases, which is consistent with the results in Fig. 4(b). The exact underlying dynamics remains further exploration, which might be related to the laser induced quasi-particles in the microwave cavity [S3, S4].

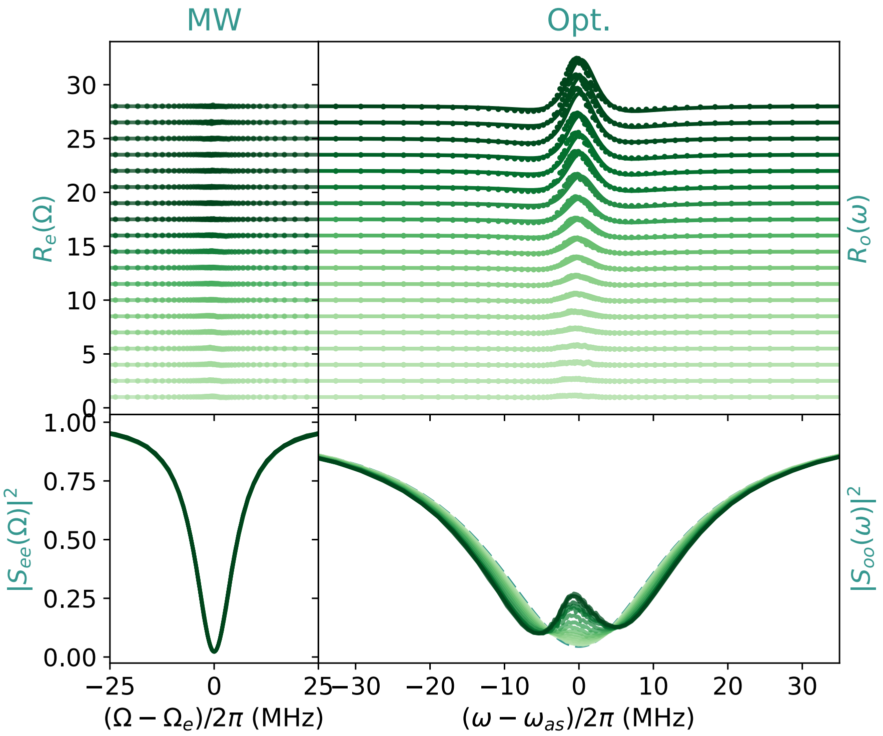

FIG. S7. **Coherent stationary response of microwave and anti-Stokes mode in the symmetric mode configuration ($J_{\mathrm{s/as}}=0$) at different C as in Fig. 2 and 3.**

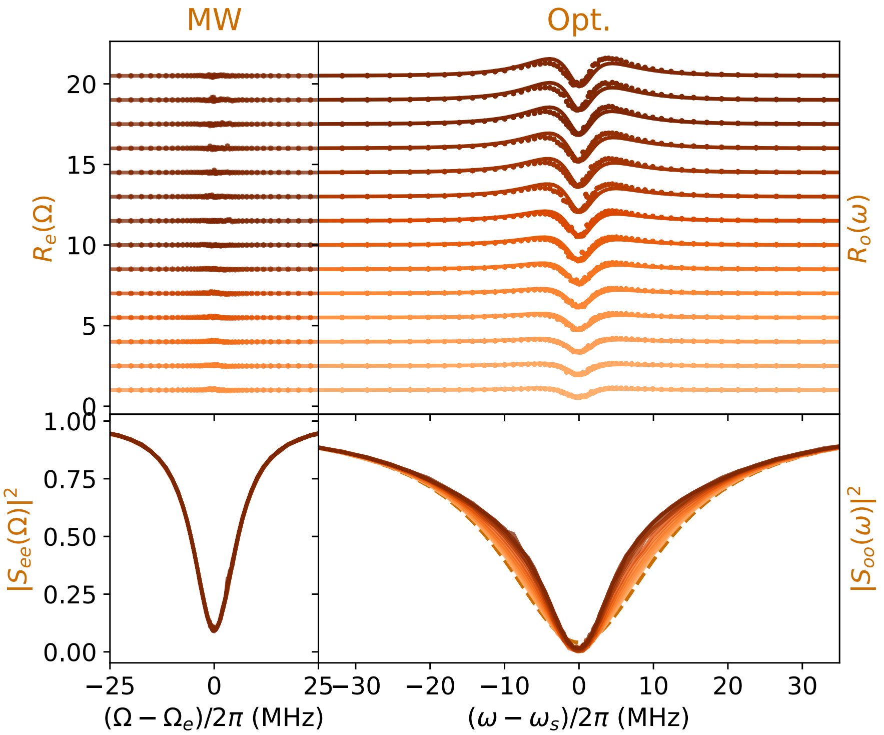

FIG. S8. **Coherent stationary response of microwave and optical Stokes mode in the symmetric mode configuration ($J_{\mathrm{s/as}}=0$) at different C as in Fig. 2 and 3.**

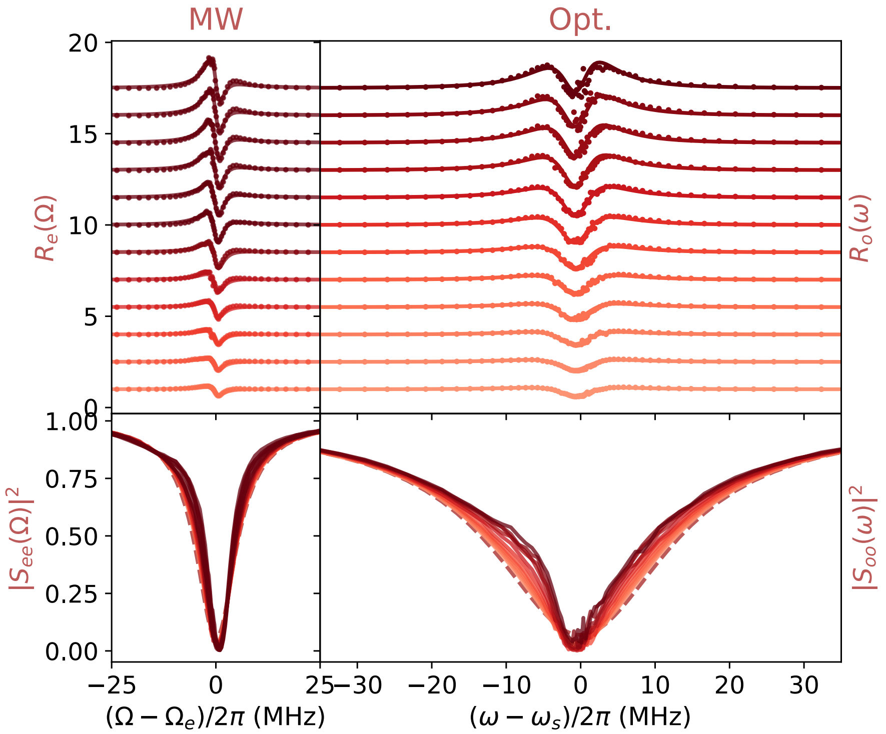

FIG. S9. **Coherent stationary response of microwave and Stokes mode in the Stokes case ($J_s = 0$) at different C as in Fig. 2 and 3.**

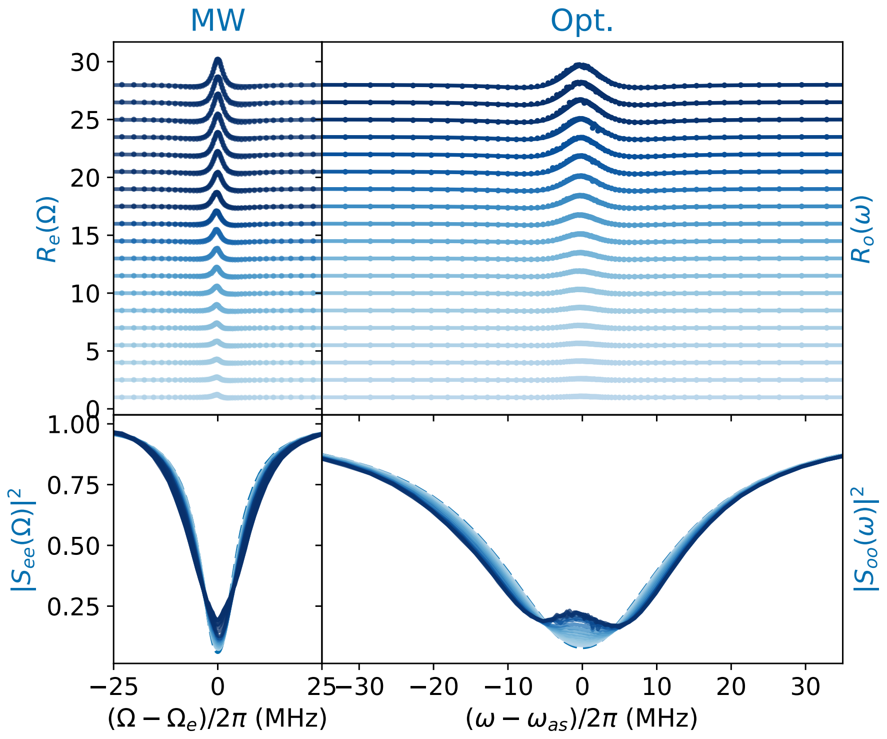

FIG. S10. **Coherent stationary response of microwave and anti-Stokes mode in the anti-Stokes case ($J_{as} = 0$) at different C as in Fig. 2 and 3.**

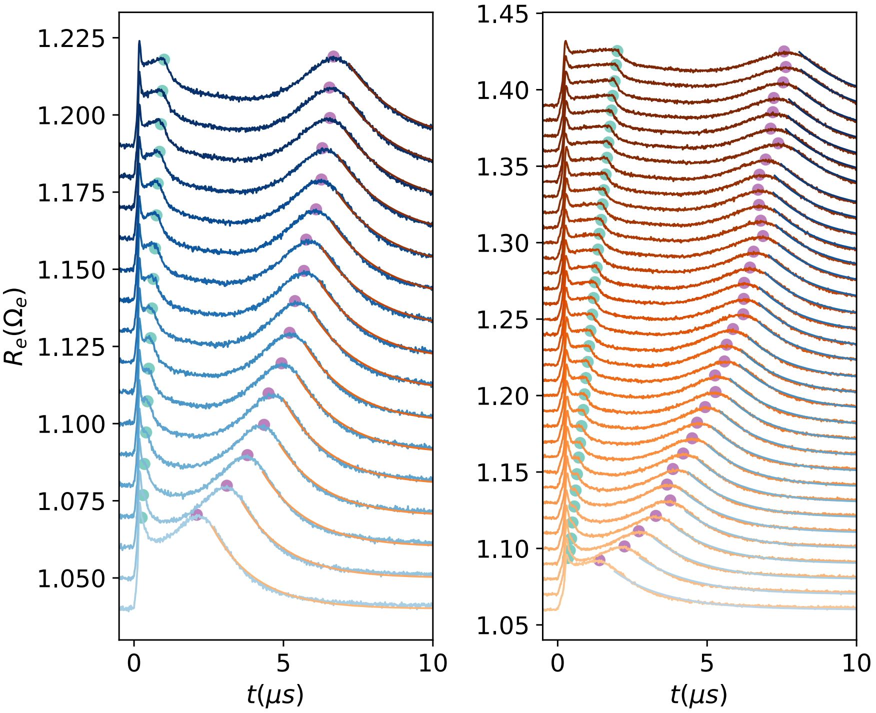

FIG. S11. **Instantaneous coherent microwave response in the symmetric case ($J_{\mathrm{s/as}} = 0$) with different pulse length as in Fig. 4(c).** The resonant ($\Omega_e = FSR$) and off-resonant ($\Omega_e \neq FSR$) cases are shown in the left and right panels. The corresponding pulse end and the bounce of the delayed back-action are indicated with green and purple dots, where the time difference is t_{ex}. The mean lifetime τ_{ex} is fitted from exponential decay of $R_e(\Omega_e)$ after the bounce, shown as orange and blue curves in the left and right panels.

[S1] Y. Xu, A. A. Sayem, C.-L. Zou, L. Fan, R. Cheng, and H. X. Tang, Photorefraction-induced Bragg scattering in cryogenic lithium niobate ring resonators, Optics Letters **46**, 432 (2021).

[S2] L. Qiu, G. Huang, I. Shomroni, J. Pan, P. Seidler, and T. J. Kippenberg, Dissipative Quantum Feedback in Measurements Using a Parametrically Coupled Microcavity, PRX Quantum **3**, 020309 (2022).

[S3] J. D. Witmer, T. P. McKenna, P. Arrangoiz-Arriola, R. V. Laer, E. A. Wollack, F. Lin, A. K.-Y. Jen, J. Luo, and A. H. Safavi-Naeini, A silicon-organic hybrid platform for quantum microwave-to-optical transduction, Quantum Science and Technology **5**, 034004 (2020).

[S4] M. Mirhosseini, A. Sipahigil, M. Kalaee, and O. Painter, Superconducting qubit to optical photon transduction, Nature **588**, 599 (2020).
